# Unilateral acute maculopathy associated with adult onset hand, foot and mouth disease: case report and review of literature

**DOI:** 10.1186/s12348-015-0034-3

**Published:** 2015-02-01

**Authors:** Rupesh Agrawal, Kanchan Bhan, Kam Balaggan, Richard WJ Lee, Carlos E Pavesio, Peter KF Addison

**Affiliations:** Moorfields Eye Hospital, NHS Foundation Trust, 162 City Road, London, EC1V 2PD UK; Biomedical Research Centre, Moorfields Eye Hospital NHS Foundation Trust and UCL Institute of Ophthalmology, 162 City Road, London, EC1V 2PD UK; National Healthcare Group Eye Institute, Tan Tock Seng Hospital, 11 Jalan Tan Tock Seng, Singapore, 308433 Singapore; University Hospitals Bristol NHS Foundation Trust, Upper Maudlin Street, Bristol, BS2 8HW UK

**Keywords:** Acute maculopathy, Posterior uveitis, Coxsackie virus, Hand, foot and mouth disease (HFMD), Autofluorescence

## Abstract

**Background:**

Acute maculopathy is a rare condition of unknown aetiology and Coxsackie virus is known to be associated with this macular chorioretinitis.

**Findings:**

We report a case of acute unilateral maculopathy in a 35-year-old woman with concurrent hand foot and mouth disease. Furthermore, we display multimodal imaging (colour fundus photographs, autofluorescence, spectral domain ocular coherence tomography, fluorescein angiography and indocyanine green angiography) charting the course of the disease. The source of the virus was thought to be the patient's child. Empirical treatment with oral corticosteroids was commenced and the inflammation resolved, leaving a residual macular scar.

**Conclusions:**

We present this case combined with the review of literature of adult onset Coxsackie-virus-associated retinitis. This case reiterates the fact that Coxsackie virus is an uncommon but important consideration in the differential diagnosis of chorioretinitis and posterior uveitis with atypical retinopathy.

## Findings

### Introduction

A taxonomically diverse group of viral agents are known to affect the posterior segment of the eye, either as a primary infection or secondary to vascular sequelae [1]. Viruses may invade the eye by direct inoculation or through metastatic spread via blood or nerves. Herpes viruses are the commonest viruses causing posterior segment infections [2]. Coxsackie virus, one of the enteroviruses, is also known to infect the eye and can lead to conjunctivitis, keratoconjunctivitis and uveitis.

Coxsackie virus can lead to hand, foot and mouth disease (HFMD) which is a highly contagious condition predominantly affecting children [3]. It is characterised by maculopapular or vesicular eruptions on the hands, feet and inside the mouth. It usually follows a benign and self-limiting course. Severe systemic complications include encephalitis, meningitis, pulmonary edema and myocarditis. There are limited case reports of Coxsackie virus causing infection in adults and sporadic reports of Coxsackie virus leading to ocular complications in adults [4].

We report a case of acute maculopathy in a 35-year-old female with concurrent hand, foot and mouth disease. Multimodal imaging, including previously undescribed autofluorescence findings, displays the outer retinitis and ensuing resolution.

### Case report

**Figure 1:**
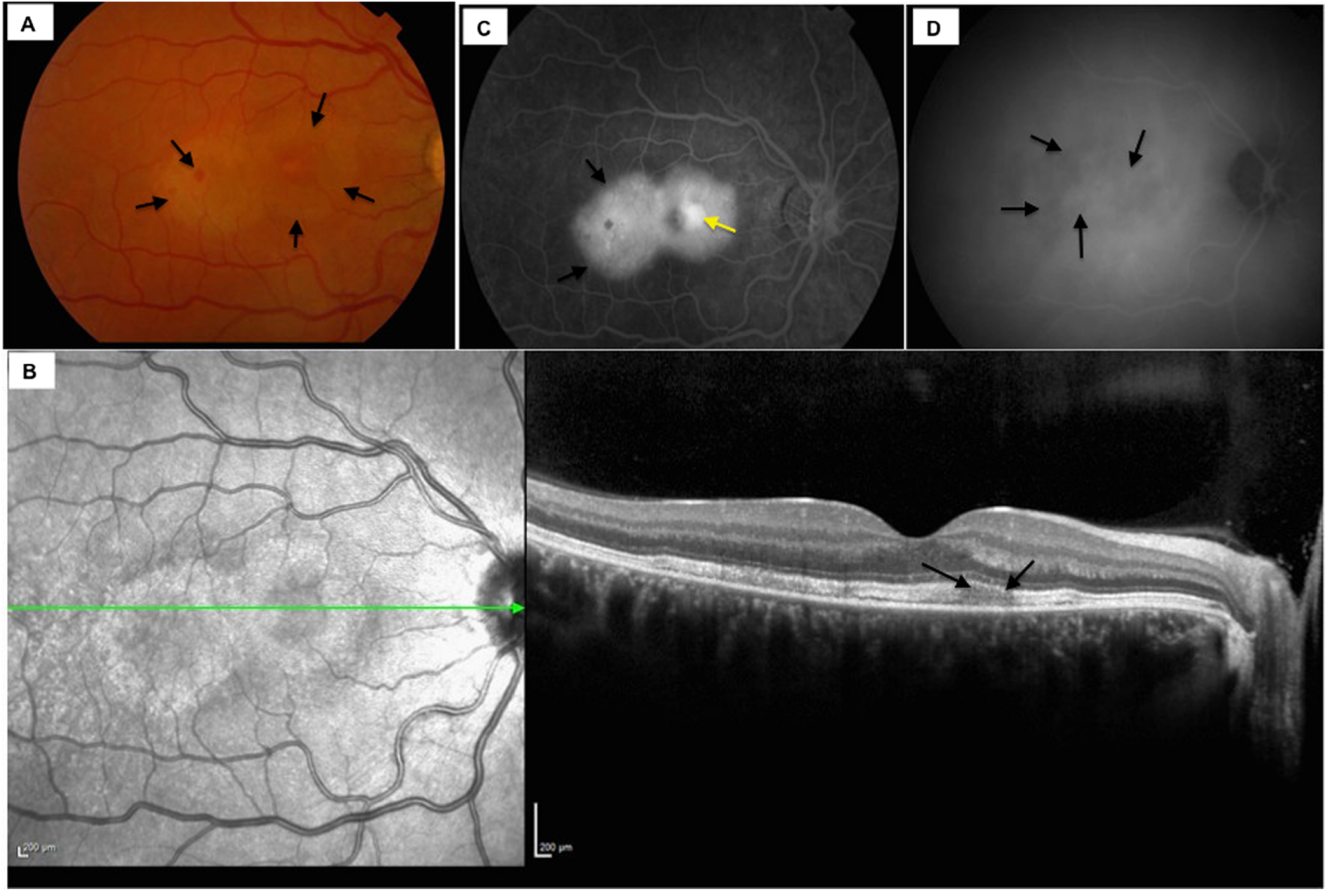
**Clinical profile and ancillary investigations at baseline.** Color fundus photography demonstrates the presence of irregular, ill defined, circular area of white grey discoloration of the right macula with intraretinal haemorrhage, intraretinal thickening and retinal pigmentary changes temporal to the fovea **(A)**. Infrared imaging showing the presence of a figure of eight or dumb-bell-shaped lesion with disruption of the inner segment-outer segment (IS-OS) junction and hyperreflective debris at the apical side of the retinal pigment epithelium on the corresponding spectral domain ocular coherence tomography (SD-OCT) **(B)**. Fundus fluorescein angiography showing a large area of intense subretinal hyperfluorescence indicating significant macular leakage **(C)** along with a small central area of pooling (yellow arrow) with no extra-macular abnormalities and no vasculitis. Indocyanine green angiography demonstrating irregular heterogenous patches of blocked fluorescence in the corresponding area **(D)**.

**Figure 2:**
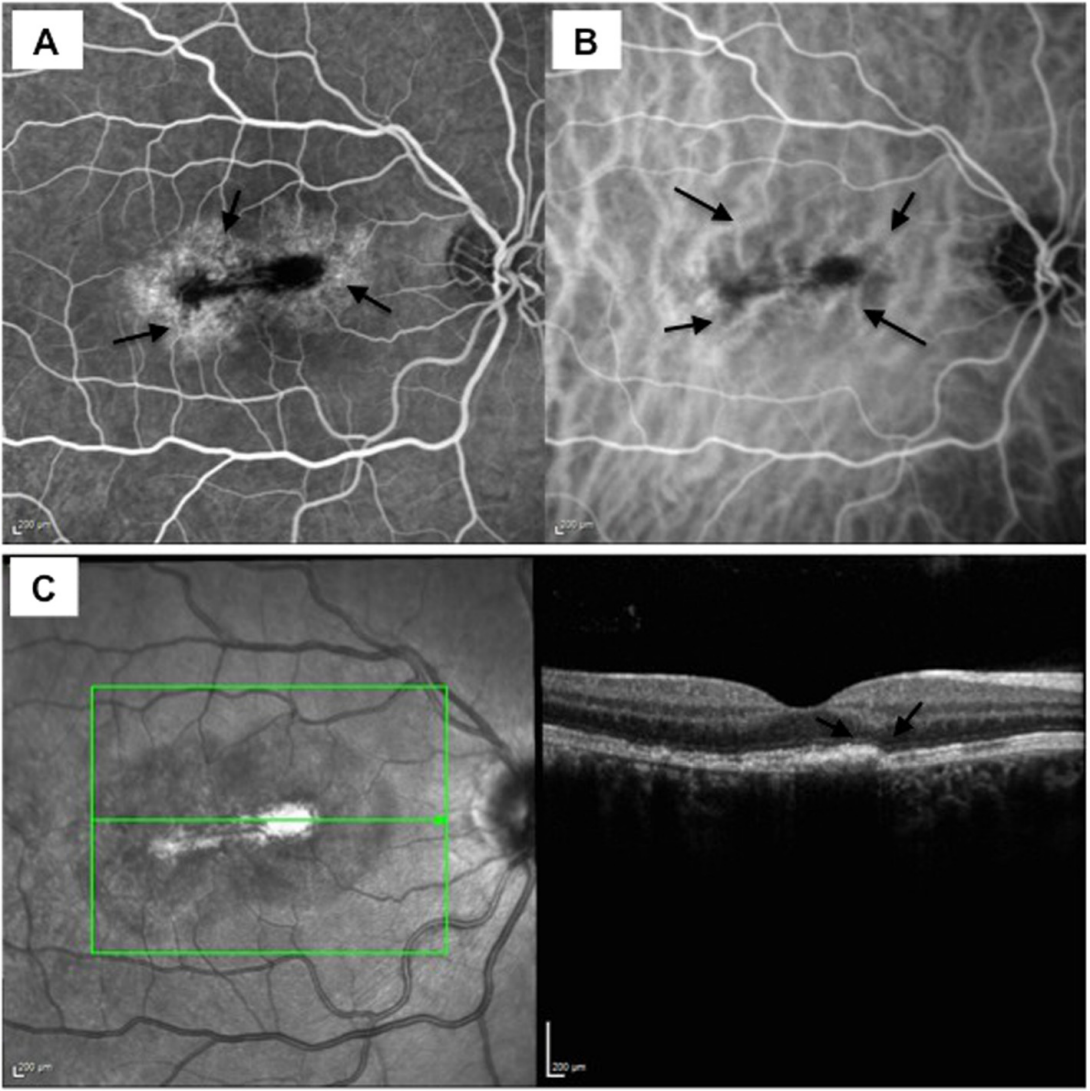
**Follow-up investigations.** Funds fluorescein angiography **(A)** demonstrates late staining with reduced leakage, and indocyanine green angiography **(B)** illustrates irregular ‘moth-eaten’ choroidal vasculature with tortous choroidal vessels around the foveal region. A residual scar remained in the right macula with intraretinal pigmentary change and resolution of subretinal fluid with a subretinal fibrotic scar on SD-OCT **(C)**. There is a presence of disrupted irregular photoreceptor layer with hyperreflective debris on the apical side of the retinal pigment epithelium.

**Figure 3:**
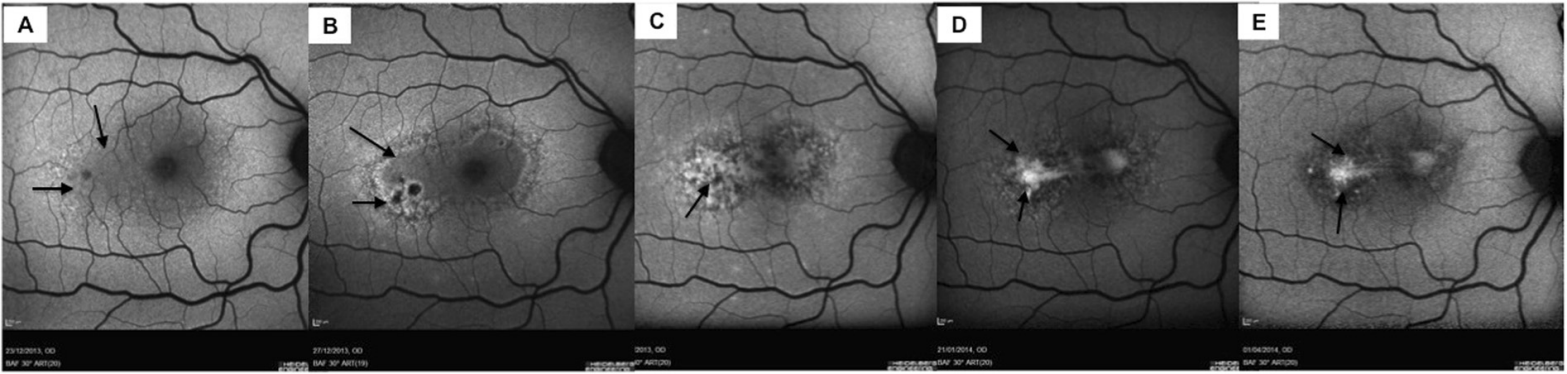
**Autofluorescence images.** Autofluorescence at baseline revealed a irregular hypo-autofluorescent area with a strip of hyperautofluorescence at temporal edge **(A)**, which over the next 4 days became morphologically similar to a dumb-bell-shaped maculopathy with increasing hyperautofluorescence at the rim of hypo-autofluorescent lesion **(B)**. The lesion showed more stippled hyperautofluorescence at 1 month **(C)** with constricting lesion and loss of background fluorescence on subsequent follow-up at 2 months **(D)** and at 4 months **(E)**.

A 35-year-old woman presented with a 1-day history of a right central scotoma and metamorphopsia which was preceded by viral exanthem and a macular rash over the hand and in the mouth. On examination, the dermatological findings were characteristic of HFMD, and there was an associated history of contact with the virus from the patient's daughter. Visual acuity was 20/20 in both eyes with right central scotoma. Fundus examination revealed right macular intraretinal haemorrhage, intraretinal thickening and retinal pigmentary changes temporal to the fovea (Figure 1A). No signs of vasculitis or chorioretinitis were seen peripherally. The left eye revealed no abnormalities. The following day the Snellen visual acuity had dropped to 20/80 in the right eye. Infrared imaging showed the presence of a figure of eight or dumb-bell-shaped lesion with abnormalities of the inner segment-outer segment (IS-OS) junction and hyperreflective debris along the retinal pigment epithelium (RPE) on the corresponding spectral domain ocular coherence tomography (SD-OCT), (Figure 1B). Fundus fluorescein angiography (FFA) showed significant macular leakage (Figure 1C) with no extra-macular abnormalities and no vasculitis. Indocyanine green angiography (ICGA) demonstrated blocked fluorescence in the corresponding area (Figure 1D). Oral prednisolone treatment was commenced with 60 mg/day once daily dose in the morning after breakfast (1 mg/kg body weight/day). Over the next few days, the scotoma became well defined and constricted. Corticosteroid therapy was tapered by 10 mg/day every week till 20 mg/day dose and subsequently tapered by 5 mg/day every week and stopped over 2 months. Visual acuity continued to improve over successive weeks to 20/20. Dermatological lesions subsided within 2 weeks of presentation. Follow-up FFA and ICGA did not reveal the presence of active disease (Figure 2A,B). ICGA showed irregular moth-eaten appearance of the choroidal vasculature with no areas of abnormal hypercyanescence. Residual scar remained in the right macula with intraretinal pigmentary change and resolution of subretinal fluid with a subretinal fibrotic scar on SD-OCT (Figure 2C). This correlated with subjective improvement in vision and scotoma. Autofluorescence revealed a hypofluorescent lesion at base line (Figure 3A), which over the next 4 days became morphologically similar to a dumb-bell-shaped maculopathy (Figure 3B). The autofluorescence lesion started constricting on subsequent follow-up (Figure 3C,D,E).

### Discussion

Coxsackie virus infection has been implicated in vitritis and posterior uveitis. Concurrent systemic infection, decreasing titers of B3 and B4 Coxsackie virus with resolving systemic and ocular disease has been reported [5,6]. There are very few cases of adult onset Coxsackie virus infection with concurred outer retinitis reported in the literature [5,6,2,7]. Likewise, unilateral acute idiopathic maculopathy has been associated with systemic comorbid conditions like orchitis and epididymitis suggesting either direct viral infection or an autoimmune response associated with viral infection [8].

An overview of the existing literature on the adult cases with possible Coxsackie virus related to outer retinitis or uveoretinitis is presented in Table 1. Our understanding of the disease progression and process has evolved with the application of constantly emerging technologies. In the reported case, we used different non-invasive and invasive imaging modalities such as color fundus photo, autofluorescence, SD-OCT, fundus fluorescein angiogram and indocyanine green angiogram to represent the changes in the macular lesion. Fundus photographs revealed irregular, circular area of mild white grey discoloration of the central macula which was gradually replaced by granular hyperpigmentation of the retina and retinal pigment epithelium as the disease progressed though the visual acuity improved. Retinal thinning and irregularity of the outer photoreceptor layer were the striking features on SD-OCT with the disease progression. However, outer photoreceptor layer appear to be normalising over the course of the disease and associated with improvement in visual acuity. Autofluorescence as a non-invasive tool corresponded to the clinically significant lesion on color fundus photographs. Characteristically, the macular lesion showed a complex heterogenous pattern of hyper- and hypo-autofluorescence with a clear demarcation line between hypo- and hyperautofluorescence. With resolution of the disease and associated with improvement in visual acuity, the lesion showed more hypo-autofluorescence with decreasing patches of hyperautofluorescence which was suggestive of loss of retinal pigment epithelium. There are no cases describing the use of autofluorescence as a follow-up tool in monitoring the associated retinal lesions. In this report, we have described the application of this non-invasive tool from the acute to resolved stage of the condition, suggesting that, similar to other conditions which affect the retinal pigment epithelium, autofluorescence has a role in monitoring disease progression [9].

**Table 1:** **Head-to-head comparison of case reports of Coxsackie-virus-associated retinopathy in the world literature**

**References**	**Age/gender/eye**	**Morphology**	**Steroid treatment**
Hirakata et al. [10]	30/F/LE	Chorioretinitis	No
Forster et al. [5]	29/F/LE	Papillitis, retinal vasculitis, mid peripheral exudates in the retina, anterior chamber infiltrates	No
Kadrmas and Buzney [6]	34/F/BE	Parafoveal exudates with midperipheral confluent exudates	Yes
Takeuchi et al. [2]	34/M/BE	Uveoretinitis, retinal exudates and retinal vasculitis	No
Haamann et al. [7]	36/M/RE	Focal outer retinitis	No
Vaz-Pereira et al. [11]	31/M/RE	Macular neurosensory detachment	No
Demirel et al. [12]	30/M/RE	Outer retinitis	No
Jung et al. [13]^a^	27/F	Subfoveal exudative retinal detachment	No
	30/M	Irregular, circular areas of mild white grey discoloration of central macula	No
	31/M	Irregular, circular areas of mild white grey discoloration of central macula	No
	52/M	Irregular, circular areas of mild white grey discoloration of central macula	No

F - Female, LE - left eye, RE - right eye, BE - both eyes. ^a^Reported four cases, table list all the four cases separately/eyes are not specified in their case series but all four cases were unilateral.

Corticosteroids were administered in our patient given the rapid deterioration in vision, and there have been conflicting reports in the literature on the use of corticosteroids in unilateral acute idiopathic maculopathy with possible viral etiology. Our experience highlights that there was no progression of the viral retinopathy after administration of corticosteroids and also there was rapid recovery of vision.

### Conclusion

This case reiterates the fact that Coxsackie virus is an uncommon but important consideration in the differential diagnosis of chorioretinitis and posterior uveitis with atypical retinopathy. A careful history of dermatological manifestations and appropriate examination should be undertaken as well as ascertainment of contact history of those with characteristic HFMD signs. In this case, autofluorescence was a useful non-invasive monitoring tool in monitoring of this outer retinal disease, and vision improved dramatically following systemic corticosteroid treatment.

### Consent

The patient has given consent for the report to be published.

## Abbreviations

FFA: Fundus fluorescein angiogram
HFMD: Hand foot mouth disease
ICGA: Indocyanine green angiogram
IS-OS: Inner segment-outer segment junction
SD-OCT: Spectral domain optical coherence tomography
RPE: Retinal pigment epithelium

## References

[1] Newman H, Gooding C (2013). Viral ocular manifestations: a broad overview. Rev Med Virol.

[2] Takeuchi M, Sakai J, Usui M (2003). Coxsackievirus B4 associated uveoretinitis in an adult. Br J Ophthalmol.

[3] Xing W, Liao Q, Viboud C, Zhang J, Sun J, Wu JT, Chang Z, Liu F, Fang VJ, Zheng Y, Cowling BJ, Varma JK, Farrar JJ, Leung GM, Yu H (2014). Hand, foot, and mouth disease in China, 2008-12: an epidemiological study. Lancet Infect Dis.

[4] Kaminska K, Martinetti G, Lucchini R, Kaya G, Mainetti C (2013). Coxsackievirus A6 and hand, foot and mouth disease: three case reports of familial child-to-immunocompetent adult transmission and a literature review. Case reports in dermatology.

[5] Forster W, Bialasiewicz AA, Busse H (1993). Coxsackievirus B3-associated panuveitis. Br J Ophthalmol.

[6] Kadrmas EF, Buzney SM (1999). Coxsackievirus B4 as a cause of adult chorioretinitis. Am J Ophthalmol.

[7] Haamann P, Kessel L, Larsen M (2000). Monofocal outer retinitis associated with hand, foot, and mouth disease caused by coxsackievirus. Am J Ophthalmol.

[8] Huemer HP, Larcher C, Kirchebner W, Klingenschmid J, Gottinger W, Irschick EU (1996). Susceptibility of human retinal pigment epithelial cells to different viruses. Graefes Arch Clin Exp Ophthalmol.

[9] Sepah YJ, Akhtar A, Sadiq MA, Hafeez Y, Nasir H, Perez B, Mawji N, Dean DJ, Ferraz D, Nguyen QD (2014). Fundus autofluorescence imaging: fundamentals and clinical relevance. Saudi J Ophthalmology.

[10] Hirakata K, Oshima T, Azuma N (1990). Chorioretinitis induced by coxsackievirus B4 infection. Am J Ophthalmol.

[11] Vaz-Pereira S, Macedo M, De Salvo G, Pal B (2014). Multimodal imaging of exudative maculopathy associated with hand-foot-mouth disease. Ophthalmic Surg Lasers Imaging Retina..

[12] Demirel S, Batioglu F, Ozmert E, Batioglu F (2014). Unilateral acute maculopathy related to hand, foot, and mouth disease: OCT and fluorescein angiography findings of a very rare disease. Eur J Ophthalmol.

[13] Jung CS, Payne JF, Bergstrom CS, Cribbs BE, Yan J, Hubbard GB, Olsen TW, Yeh S (2012). Multimodality diagnostic imaging in unilateral acute idiopathic maculopathy. Arch Ophthalmol.

